# Challenges in pancreatic adenocarcinoma surgery - National survey and current practice guidelines

**DOI:** 10.1371/journal.pone.0173374

**Published:** 2017-03-07

**Authors:** Sameer A. Dhayat, Philip Mirgorod, Christina Lenschow, Norbert Senninger, Christoph Anthoni, Thorsten Vowinkel

**Affiliations:** Department of General and Visceral Surgery, University Hospital Muenster, Muenster, Germany; University of Nebraska Medical Center, UNITED STATES

## Abstract

**Background:**

Pancreatic ductal adenocarcinoma (PDAC) remains one of the most deadly cancers in Europe and the USA. There is consensus that radical tumor surgery is the only viable option for any long-term survival in patients with PDAC. So far, limited data are available regarding the routine surgical management of patients with advanced PDAC in the light of surgical guidelines.

**Methods:**

A national survey on perioperative management of patients with PDAC and currently applied criteria on their tumor resectability in German university and community hospitals was carried out.

**Results:**

With a response rate of 81.6% (231/283) a total of 95 (41.1%) participating departments practicing pancreatic surgery in Germany are certified as competence and reference centers for surgical diseases of the pancreas in 2016. More than 95% of them indicate to carry out structured and interdisciplinary therapies along with an interdisciplinary pre- and postoperative tumor board. The majority of survey respondents prefer the pylorus-preserving partial pancreatoduodenectomy (93.1%) with standard lymphadenectomy for cancer of the pancreatic head. Intraoperative histological evaluation of the resection margins is used regularly by 99% of the survey respondents. 98.7% of survey respondents carry out partial or complete vein resection, 126 respondents (54.5%) would resect tumor adjacent arteries, and 102 respondents (44.2%) would perform metastasectomy if complete PDAC resection (R0) is possible.

**Conclusion:**

Evidence-based and standardized pancreatic surgery is practiced by a large number of hospitals in Germany. However, a significant number of survey respondents support an extended radical tumor resection in patients with advanced PDAC even when not indicated by current clinical guidelines.

## Introduction

Pancreatic ductal adenocarcinoma (PDAC) is currently the fourth leading cause of cancer related death in western countries, and it is expected to rise to second place behind lung cancer until 2030.[1] In contrast to stable or declining trends for most cancers in Europe since 2009, PDAC shows an increase of 3.6% and 5.2% for men and women, respectively, with predicted death rates of 8.2 and 5.6/100 000 corresponding to 85 300 total deaths in 2015. In Germany low 5-year overall survival rates of 6% for men and 8% for woman are accompanied by similar incidence and mortality rates of about 16 000 people in 2012, making long-term survival rather exceptional.[2, 3] The poor prognosis of PDAC is mainly attributed to rapid disease progression, late diagnosis at advanced unresectable stages, and poor response to current (neo-) adjuvant or palliative regimens.

Oncological surgery with tumor extirpation as the only curative treatment option of PDAC at early stages, so far, is possible in only 15% of PDAC patients with 5-year survival rates below 20%.[4] To date, national and international guidelines for the treatment of PDAC patients differentiate between resectable, borderline resectable, locally advanced unresectable, and metastatic PDAC.[5–7] However, there is a wide range of definitions available for borderline PDAC resectability.

The aim of this study is to generate data regarding the routine surgical management of patients with PDAC in Germany. On the basis of the revised clinical practice guidelines for PDAC surgery of the American NCCN (National Comprehensive Cancer Network) in 2015, the European ESMO (European Society for Medical Oncology) in 2015, and the German AWMF (Association of Scientific Medical Societies in Germany) in 2013, we carried out a national survey on perioperative management of patients with PDAC and currently applied criteria on their tumor resectability in German university and community hospitals.[5–7] In this context, the daily practice with borderline tumors was of particular interest.

## Methods

### Study design

A national anonymized survey on surgical management of patients with PDAC in 283 German university and community hospitals was performed between November 2015 and January 2016. Questionnaires, consisting of 12 questions with 1 multiple-choice question, 10 yes/no-questions, and one open question were sent out to all German hospitals reaching a threshold of twelve pancreatic resections per year as documented in the online database www.weisse-liste.de. The addressed heads of the different surgical departments were asked for the number of PDAC surgeries per year, certification as competence and reference center for surgical diseases of the pancreas, implementation of pathways incorporating structured and interdisciplinary therapies along with pre- and postoperative interdisciplinary tumor boards, structured pathways for postoperative clinical treatment, in-house follow-up care, local criteria for tumor resectability with consecutive procedures in advanced PDAC, and implementation of an intraoperative histological evaluation of the resection margins (Table 1). Specifically, they were asked for their decision in case of a preoperatively unknown, but technically resectable distant metastasis during the surgical exploration and pancreatic resection. According to Meguid *et al*, an annual institution resection volume of 19 or more pancreatic resections was defined as high-volume center.[8] As this survey does not involve clinical research on patients but focuses on quality data of participating survey respondents, the Ethics Commission notes that neither consent nor ethics committee approval is required (Ethics Commission, University Muenster, Az: 2016-515-f-N).

**Table 1:** Questionnaire consisting of twelve question blocks with a total of 34 parameters.

**1**	**Name of hospital, chief, location, head of pancreatic surgery**	**Free text**
	**Specialized department for pancreatic surgery**	**Yes / No**
**2**	**Pancreatic procedures in general / head resections/ other**	**<12; <20; <50; <100; >100**
**3**	**DGAV or DKG (OnkoZert) certification / other certification**	**Yes / No / Free text**
**4**	**Pylorus-preserving- pancreatoduodenectomy / pancreatojejunostomy / pancreatogastrostomy / distal pancreatectomy**	**Yes / No / Occasionally**
**5**	**Presentation in pre- / postoperative tumorboard**	**Yes / No / Occasionally**
**6**	**Intraoperative histological evaluation of the resection margins**	**Yes / No**
**7**	**Intraoperative decision for preoperatively unknown, but technically resectable liver metastasis with estimated R0 / R1 / R2 resection**	**Yes / No**
**8**	**Intraoperative decision for preoperatively unknown, but technically resectable peritoneal metastasis with estimated R0 / R1 / R2 resection**	**Yes / No**
**9**	**Intraoperative decision for venous infiltration with estimated R0 / R1 / R2 resection**	**Yes / No**
**10**	**Intraoperative decision for arterial infiltration with estimated R0 / R1 / R2 resection**	**Yes / No**
**11**	**Decision if guidelines do not recommend continuation of resection**	**Free text**
**12**	**Clinical pathway / in-house follow-up care / guideline audit**	**Yes / No**

### Statistical analysis

All returning questionnaires were collected by our study nurse at the Department of General and Visceral Surgery of the University Hospital Muenster. Statistical data analysis was performed in cooperation with our Institute of Biostatistics and Clinical Research using SPSS^®^ Statistics Version 22 (IBM Corp. Armonk, NY) for Windows^®^. We used the Fisher two-tailed exact test and whenever appropriate the χ^2^ test for contingency tables. All of the variables were dichotomized. *P* value <0.05 was considered to be statistically significant.

## Results

### Survey respondents

A total of 231 of 283 questionnaires that were sent out were completed and returned. This corresponds to a response rate of 81.6%. 11.3% of the responding surgical departments with their own divisions of pancreatic surgery were located at German university hospitals and 88.7% at German community hospitals. In 2014, pancreatic surgery procedures were performed in more than 100 patients per year by 16 departments, in 50 to 100 patients per year by 23 departments, in 20 to 50 patients per year by 102 departments, in 12 to 20 patients per year by 55 departments, and in less than 12 patients per year by 10 responding surgical departments. Pancreatoduodenectomy in particular was performed in most surgical institutions of survey respondents (97%) using the pylorus-preserving partial pancreatoduodenectomy (PPPD) by Traverso—Longmire or the Classic (Kausch-)Whipple pancreatoduodenectomy (CWPD). Pancreatic head resections by PPPD was preferred by 215 (93.1%) survey respondents. 189 survey participants (81.8%) preferred the pancreatojejunostomy technique over pancreatogastrostomy (27.3%). In patients with pancreatic body or tail pathologies, 224 survey respondents (97%) would perform a distal pancreatectomy.

### Capacity levels, case load and certification

**Fig 1:**
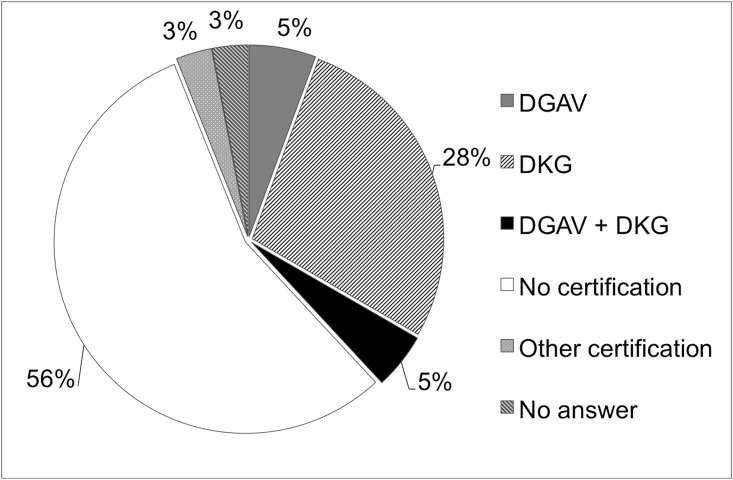
Certification status. Certification of survey respondents as competence and reference center for surgical diseases of the pancreas.

Higher capacity levels corresponded with higher case load (*p*< 0.0001), that correlated with certification status as competence and reference center for surgical diseases of the pancreas (*p*< 0.0001), tested and awarded among others by the German Association for General and Visceral Surgery (DGAV) or the German Cancer Association (DKG). Overall 75 survey respondents (32.5%) were certified by the DKG, 24 (10.4%) were certified by the DGAV, 7 (3%) stated to be certified by other oncological societies, 129 surgical departments (55.8%) were not certified, and 7 survey respondents (3%) did not provide any information to this topic (Fig 1). However, 96.1% of the survey respondents indicate to carry out structured and interdisciplinary therapies along with an interdisciplinary pre- and postoperative tumor board, an internal guideline audit (45%), and a standard postoperative care (86,1%) according to the AWMF guidelines.

### Surgical criteria for resectability

**Fig 2:**
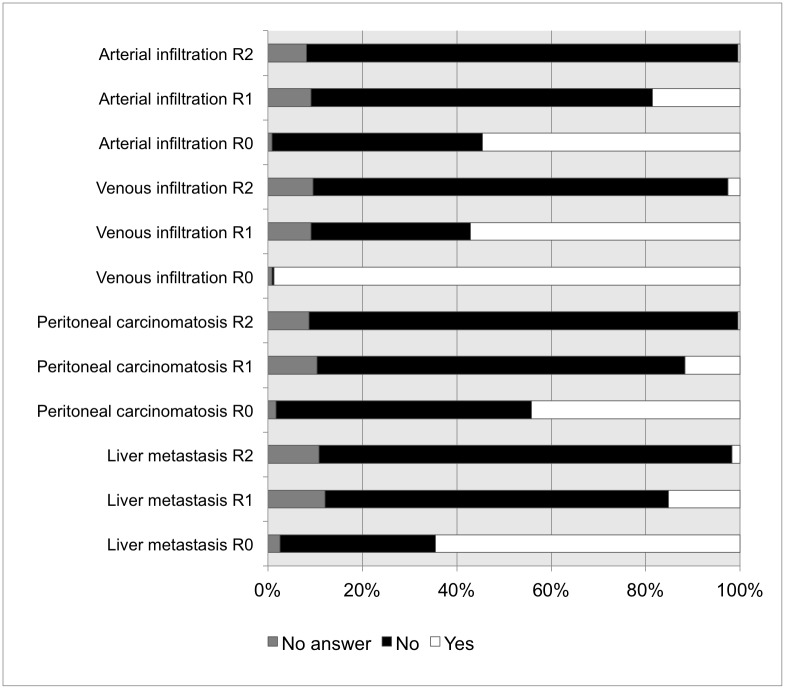
Criteria for tumor resectability. Intraoperative decision and percentages of surgical (non-) resectability in PDAC.

54.5% of the survey respondents consider the presence of peritoneal metastasis as the most important criterion for non-resectability of PDAC. However, 102 departments (44.2%) would perform metastasectomy if complete resection (R0) is possible. Microscopic residual tumor (R1) would be accepted by 26 departments (11.3%), macroscopic residual tumor (R2) by 1 department (0.4%), depending on the individual case (Fig 2). Intraoperative histological evaluation of the resection margins is used regularly by 99% of the responding surgical departments. In case of technically resectable liver metastasis, 64.5% of the survey respondents would remove them if R0 is possible. Some survey respondents would carry out resection of liver metastasis, even if only R1 (15.2%) or R2 (1.7%) results are possible. PDAC infiltration of adjacent arteries is regarded as resectable by 126 respondents (54.5%) if R0 is possible. Depending on the individual case, some departments would perform arterial resection, even if only R1 (18.6%) or R2 (0.4%) results are possible. In case of singular venous PDAC infiltration, a majority of 98.7% of survey respondents carry out partial or complete vein resection in curative intention, whereas 57.1% / 2.6% would even accept a possible R1 / R2 situation, respectively.

A total of 9 different answers of the survey respondents (81.2%) can be categorized with regard to the open question of the survey about their approach to advanced tumor, in which the guidelines recommend no further resection: Termination of further tumor resection in accordance with the guidelines in 31.2%, case-dependent individual decision of the surgeon in 29.0%, consultation of the surgical head of the department in 7.8%, interdisciplinary consultation of e.g. oncologist or pathologist in 3.9%, and continuation of tumor resection in 1.3%.

Subgroup analyses reveal that a case load at high volume centers with more than 20 pancreatoduodenectomies in 2014 has influence on treatment decisions. The assessment of technically resectable distant metastasis and local vascular tumor infiltration differed significantly between survey respondents of high and low volume hospitals with resection rates of 50% *versus* 12.9% (*p* = 0.023) for liver metastasis, and resection rates of 92.3% *versus* 43.3% (*p* = 0.007) for venous PDAC infiltration.

## Discussion

Radical surgery is still regarded as the best option for potentially curative treatment and long-term survival in patients with PDAC.[9, 10] However, the resection rate with curative intent remains less than 20%.[1] Redefining of locally advanced non-metastatic PDAC into borderline resectable disease and non-resectable disease is an approach to maximize the option of a personalized curative attempt for PDAC patients who were previously considered to have unresectable disease.[11] To improve the rate of complete resection of the primary tumor to microscopically negative margins, an expert consensus group has developed criteria to define tumour resectability that were adopted to current national clinical practice guidelines.[5–7, 10–13] These imaging-based criteria for borderline resectability include no distant mestastasis with safe resection and reconstruction of (1) short segment venous involvement of the superior mesenteric vein (SMV) or portal vein (PV), and (2) arterial segment involvement of the superior mesenteric artery (SMA) or celiac trunk <180° or gastroduodenal artery encasement up to the hepatic artery with no extension to the celiac axis. Although computed tomography or magnetic resonance imaging are able to determine PDAC non-resectability with a high positive predictive value of >90%, their predictive value to affirm resectability in less than 50% is insufficient.[14] Furthermore, the lack of consensus with regard to objective definition and therapeutic algorithm put borderline PDAC at risk of being classified falsely as unresectable disease stage.

In this national survey, we demonstrate and analyse the routine surgical management of patients with PDAC in 231 German university and community hospitals practicing pancreatic surgery. This high response rate of 81.6% reflects the actual surgical treatment of PDAC in Germany and its accordance with the current consensus-based recommendations suggested by the clinical practice guidelines for PDAC surgery. Interestingly, it seems that different opinions on the definition to technically resectable PDAC exist. A significant number of responding departments would perform resection of preoperatively unknown, but technically resectable liver metastasis in 64.5%, resection and reconstruction of tumor-adjacent arterial encasement in 54.5%, and even metastasectomy in 44.2%, if radical tumor resection is possible. These results represent a progressive surgical approach of many survey respondents to resect advanced PDACs including potentially R0-resectable peritoneal or liver metastases in highly selected patients. Indeed, the evidence for this indication is limited. However, very recent data suggest improved survival for patients undergoing synchronous resections of PDAC and single liver or pulmonary metastases.[15–17] Obviously, the resection of the primary tumor and synchronous liver or peritoneal metastases is not recommended by current national and international guidelines for the treatment of PDAC. In such palliative cases, chemotherapeutic regimes with FOLFIRNOX or gemcitabine and nab-paclitaxel have been established showing increased overall survival with a median of up to 11 months. With increasing safety of operations and persisting unsatisfying oncologic outcome in PDCA patients even in early stages, extension of localized approaches in pancreatic surgery, partially combined with neo-adjuvant treatment is documented in the literature. There is mounting evidence that high volume pancreas centers support the potential benefit of surgical intervention with macroscopically complete resection in highly selected patients with locally advanced or metastatic PDAC. In recent years, small retrospective studies and case reports including highly selected patients in good general condition suggest that extended resections of PDAC and even of distant diseased lymph nodes and liver metastases can be performed safely with improved survival.[15, 18–26] However, randomized controlled trials on this issue are lacking so far. In contrast, there is wide acceptance that mesenterico-portal resection ranging from partial venous excision to segmental resection is recommended in PDAC with single venous infiltration allowing safe resection and reconstruction.[27–29]

Overall, there is broad international variation of borderline PDAC treatment. The great majority of survey respondents prefer the direct operative exploration for potential resection *versus* 0.4% suggesting a downstaging by neoadjuvant therapy. Recently, a systematic review by Tang *et al*. including trials with prospective design could reveal similar R0 resection and survival rates in borderline resectable PDAC patients after neoadjuvant therapy and in resectable PDAC patients, much higher than those in unresectable tumor patients. It is noteworthy that 63% of PDAC patients initially staged as borderline resectable could be resected successfully after neoadjuvant therapy with a median survival amounted to 25.9 months (range 21.1–30.7).[30] Common definition and treatment algorithm for borderline resectable PDAC should be applied in future trials.

As expected, the decision of survey respondents for radical surgical resection even in advanced tumor stages with potentially resectable venous or arterial infiltration and even solitairy R0 resectable hepatic or peritoneal metastasis correlates with the establishment of experienced high-volume centers. Several studies could demonstrate that pancreatectomy performed at high volume PDAC treatment centers by high-volume surgeons improves short- and long-term outcomes.[31, 32] However, therapy strategies currently not recommended by scientific evidence should be offered to PDAC patients with advanced tumor stage only within controlled clinical trials.

Pancreatoduodenectomy, preferentially PPPD, is performed in German hospitals of all capacity levels, ranging from university hospitals to community hospitals with caseloads varying from under twelve to over a hundred of annual pancreatoduodenectomies. However, less than 42% of the responding surgical departments declare to be certified as competence center for surgical diseases of the pancreas, and only 45% have an internal guideline audit. After all, 95% of the survey respondents indicate to carry out structured and interdisciplinary therapies along with an interdisciplinary pre- and postoperative tumor board, and at least 86.1% perform standard postoperative care with implementation of structured pathways for postoperative clinical treatment, in-house follow-up care for PDAC, and guideline audits according to the national guidelines. It is well known that clinical certification by accredited expert panels with implementation of clinical pathways are associated with improved professional practice and patient outcomes as well as reduced length of hospital stay and hospital costs.[33, 34]

Although pancreatic surgery is offered by a large number of hospitals in Germany, a significant number of survey respondents support an extended radical tumor resection in patients with advanced PDAC even when not indicated by the current clinical guidelines. Surgical treatment with curative intend of patients with advanced PDAC should be recommended only in the setting of prospective trials at certified high-volume competence and reference centers for surgical diseases of the pancreas.

## Abbreviations

AWMF: Association of Scientific Medical Societies in Germany
CHA: Common hepatic artery
CWPD: Classic (Kausch-)Whipple pancreatoduodenectomy
DGAV: Deutsche Gesellschaft für Allgemein- und Viszeralchirurgie, German Association for General and Visceral Surgery
DKG: Deutsche Krebsgesellschaft, German Cancer Association
ESMO: European Society for Medical Oncology
GA: Gastroduodenal artery
ISGPS: International Study Group of Pancreatic Surgery
NCCN: National Comprehensive Cancer Network
PDAC: Pancreatic ductal adenocarcinoma
PPPD: Pylorus-preserving partial pancreatoduodenectomy by Traverso—Longmire
PV: portal vein
SMA: superior mesenteric artery
SMV: superior mesenteric vein
